# Friedreich's Ataxia Frequency in a Large Cohort of Genetically Undetermined Ataxia Patients

**DOI:** 10.3389/fneur.2021.736253

**Published:** 2021-12-09

**Authors:** Alexander F. Brown, Michael H. Parkinson, Hector Garcia-Moreno, Ese Mudanohwo, Robyn Labrum, Mary Sweeney, Paola Giunti

**Affiliations:** ^1^Ataxia Centre, Department of Clinical and Movement Neurosciences, UCL Queen Square Institute of Neurology, Queen Square, London, United Kingdom; ^2^Neurogenetics Unit, National Hospital for Neurology & Neurosurgery, University College London Hospitals, Queen Square, London, United Kingdom

**Keywords:** Friedreich's ataxia, molecular diagnostics, GAA expansion, triplet-primed PCR, long range PCR

## Abstract

**Background:** Patients with suspected genetic ataxia are often tested for Friedreich's ataxia (FRDA) and/or a variety of spinocerebellar ataxias (SCAs). FRDA can present with atypical, late-onset forms and so may be missed in the diagnostic process. We aimed to determine FRDA-positive subjects among two cohorts of patients referred to a specialist ataxia centre either for FRDA or SCA testing to determine the proportion of FRDA cases missed in the diagnostic screening process.

**Methods:** 2000 SCA-negative ataxia patients, not previously referred for FRDA testing (group A), were tested for FRDA expansions and mutations. This group was compared with 1768 ataxia patients who had been previously referred for FRDA testing (group B) and were therefore more likely to have a typical presentation. The phenotypes of positive cases were assessed through review of the clinical case notes.

**Results:** Three patients (0.2%) in group A had the FRDA expansion on both alleles, compared with 207 patients (11.7%) in group B. The heterozygous carrier rate across both cohorts was of 41 out of 3,768 cases (1.1%). The size of the expansions in the three FRDA-positive cases in group A was small, and their presentation atypical with late-onset.

**Conclusions:** This study demonstrates that FRDA is very rare among patients who were referred purely for SCA testing without the clinical suspicion of FRDA. Such cases should be referred to specialist ataxia centres for more extensive testing to improve patient management and outcomes.

## Introduction

Friedreich's ataxia (FRDA), first described by Nikolaus Friedreich in 1863, is an autosomal recessive disease in which patients develop progressive ataxia involving immobility, lack of manual dexterity, dysarthria, and hypertrophic cardiomyopathy. It primarily affects the dorsal root ganglia (DRGs) and cerebellar granule cells (1, 2). In 1996, the mutation causing this disease was mapped to an intronic locus of the *FXN* gene on chromosome 9q13, which codes for the frataxin protein, which is pathologically deficient in affected cells in FRDA patients (3). This highly conserved mitochondrial protein is involved in iron-sulphur (Fe-S) cluster assembly (4). Frataxin deficiency has been shown to cause a mitochondrial energy imbalance between respiratory chain complexes I and II in cerebellar granule neurons from a FRDA mouse model, leading to oxidative damage (5). Frataxin deficiency has also been linked with Ca^2+^ deregulation in FRDA-model heart cells (6). The mutation is, in the great majority of cases, a homozygous, unstable GAA trinucleotide expansion mutation, of between 70 and 1700 repeats (4, 7). Unaffected individuals have up to 33 repeats (8). The GAA expansion size accounts for 40% of the clinical variability in FRDA clinical symptoms (9). The greatest correlation in clinical features is with the size of the smaller of the two expansions (GAA1) (9, 10). The largest case series to date revealed an average GAA1 allele size of 648 triplet repeats, and an average GAA2 allele size of 912 repeats (9). Patients with late-onset atypical FRDA have a smaller number of repeats in the GAA1 allele, typically between 100 and 500 (10–13). Repeat numbers above normal but below the expansion threshold of around 70 repeats are classed as premutations which can expand into pathogenic mutations in future generations (14, 15). Compound heterozygosity involving a GAA expansion and a point mutation in the *FXN* gene occur in a small minority of FRDA patients (4, 16–18), and compound heterozygosity with an exonic deletion is exceedingly rare (19–21).

FRDA is the most common autosomal recessive form of ataxia. The prevalence of the condition has been estimated at between 1:20,000 and 1:125,000 in several cohorts of West European populations, and has only presented thus far in people of Eurasian and North African ethnicity (22–24). The carrier frequency in the UK population is thought to be between 1:60 and 1:110 (25). The onset of disease is usually before 25 years of age: when it occurs after this age it is known as late-onset Friedreich's ataxia [LOFA; (26, 27)]. Onset after the age of 40 is known as very late-onset Friedreich's Ataxia [VLOFA; (11)]. LOFA and VLOFA are phenotypically distinct from classical FRDA, with a milder phenotype, slower disease progression and a more variable set of symptoms. FRDA studies have shown an inverse correlation between GAA expansion length and age at onset of disease, as well as a positive correlation between the size of the expansion and the rate of progression of symptoms (11, 13, 28). In LOFA patients, the size of the GAA2 allele is more relevant to age at onset. In these cases, the GAA1 allele accounts for 62.9% of variation in age at onset, and GAA2 accounts for 15.6% (27).

Evidence of atypical, late-onset FRDA phenotypes has broadened the clinical definition of FRDA, as up to 25% of genetically diagnosed FRDA patients do not fit the diagnostic criteria originally proposed by Harding in 1981 (10, 13). Age at onset as late as 70 years of age has been described. These patients may well be misdiagnosed due to their atypical presentation (29, 30). An earlier study showed that 87% of cases of typical FRDA and 36% of patients with late-onset FRDA, or FRDA with retained reflexes tested positive for *FXN* GAA expansions, revealing the difficulty of clinically distinguishing between expansion-positive and expansion-negative ataxic individuals with similar symptoms. Only 1 out of 11 cases of ‘early-onset non-Friedreich's recessive or sporadic ataxia’ proved to have the GAA expansion (31). The common spinocerebellar ataxias (SCAs 1, 2, 3, 6, 7, 12 & 17) are within the differential diagnosis of late-onset ataxia. Therefore, we were interested to see whether FRDA was responsible for a significant proportion of patients referred to our centre for genetic testing for the common SCAs but not FRDA.

## Materials and Methods

### Case Ascertainment

A cohort of 2000 ataxic patients (group A) was identified for whom no clear genetic cause had been found, despite being referred to our specialist Neurogenetics laboratory for SCA testing. As these subjects usually had a late-onset ataxic phenotype that contrasted with the classical younger onset presentation of FRDA, they had not been screened for FRDA. Data from a second group (group B) of 1,768 ataxia patients who had been referred for FRDA testing were also collected. The patients in both groups A and B were identified and analysed as a consecutive series over 10 years from 2003 to 2013. All cases were referred to the Neurogenetics Laboratory of the UCL Institute of Neurology which is associated with the National Hospital for Neurology and Neurosurgery, Queen Square, London, UK. This is a University hospital tertiary referral centre which receives referrals locally and nationally from throughout the UK, primarily from Neurologists but also from other clinicians. All patients signed informed consent for genetic testing. The study was reviewed and approved by the UCLH Joint Research Office and deemed a service evaluation. The study was conducted following guidelines set out in the Declaration of Helsinki, 1964.

### Two-Stage PCR Process

**Figure 1:**
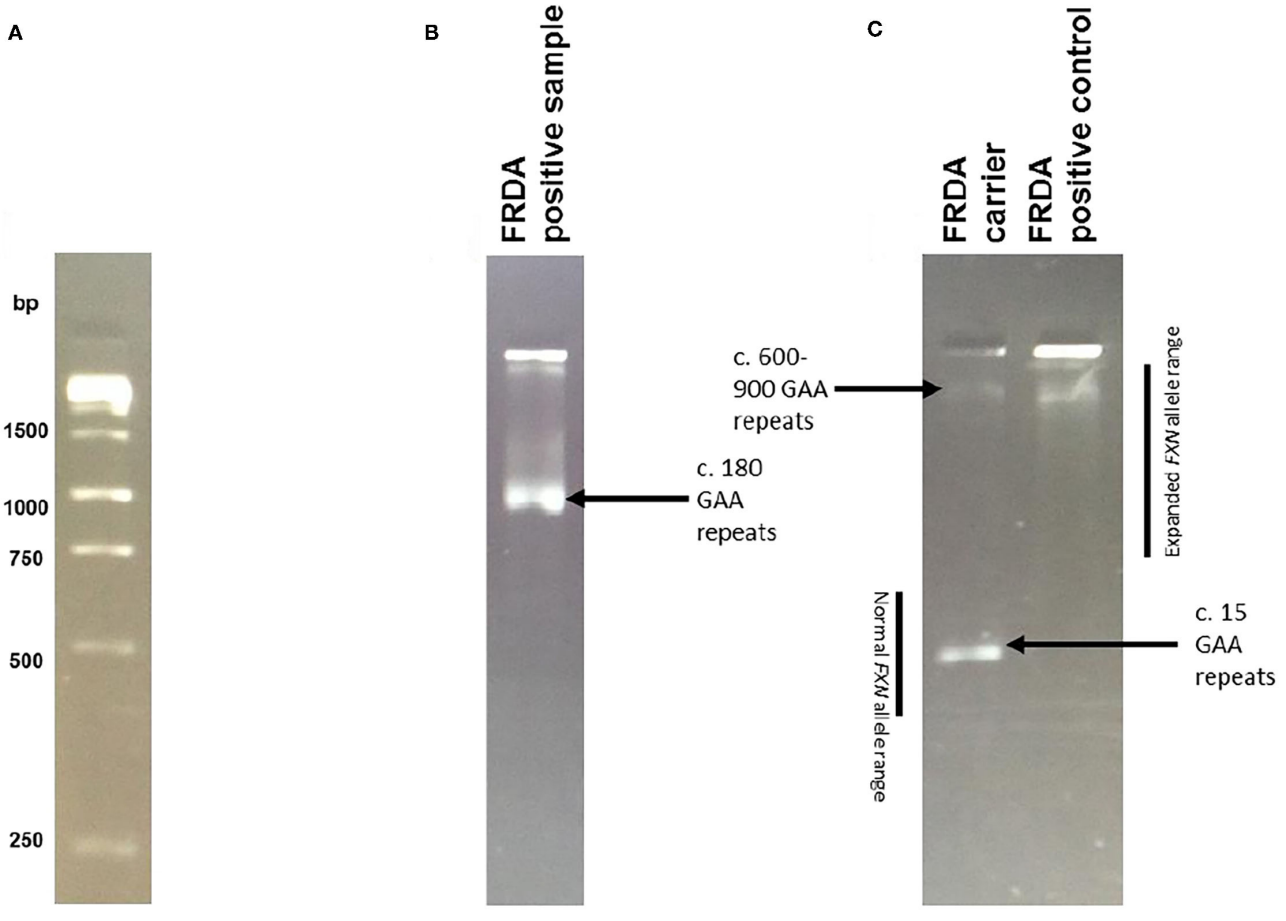
Long PCR Results. Example of results obtained by Long Range PCR, visualised on a 2% agarose gel following electrophoresis. To calculate the number of GAA repeats, the required formula was allele size = (number of base pairs-450)/3. The reasoning behind this calculation is that the synthesised PCR products contain flanking sequences totaling 450 base pairs in addition to the GAA trinucleotide repeat expansion. Normal alleles run at approximately 500 base pairs, whereas affected alleles run at approximately 720 base pairs of above. **(A)** The 1kb ladder used to calculate expansion sizes. **(B)** An example of an FRDA positive sample, with c. 180 GAA repeats. **(C)** An example of an FRDA carrier and positive control. The FRDA carrier control has one large GAA expansion of between 600 and 900 repeats and one GAA expansion of only 15 repeats, within the normal range. The FRDA positive control has two large GAA expansions of between 600 and 900 repeats.

Patient DNA samples were first subjected to the triplet-repeat primed PCR (TP-PCR) technique developed by Warner et al. (32) showing the presence of a GAA expansion on either allele of the *FXN* gene. The long-range PCR (LR-PCR) technique then determined allelic status and hence distinguished between *FXN* expansion homozygous (GAA+/+) and heterozygous (GAA+/−) individuals through observation of the DNA bands following agarose gel electrophoresis (Figure 1). Samples that displayed a homozygous *FXN* expansion were then confirmed by capillary electrophoresis, in which fragment length analysis was undertaken on an ABI (Carlsbad, CA) 3730xl automated sequencer.

### Point Mutation Screening

Point mutation sequencing was carried out on DNA from GAA+/− patients in the Merseyside and Cheshire Regional Molecular Genetics laboratory, Liverpool Women's Hospital, Liverpool, UK and the Université Libre de Bruxelles. Exons 1 to 5a were screened as this covered the coding region of the *FXN* transcript. The following primers were used to detect point mutations using the method of (33):

Exon 1: 5'-AGC ACC CAG CGC TGG AGG-3' (forward)
  5'-CCG CGG CTG TTC CCG G-3' (reverse)
Exon 2: 5'-AGT AAC GTA CTT CTT AAC TTT GGC-3' (forward)
  5'-AGA GGA AGA TAC CTA TCA CGT G-3' (reverse)
Exon 3: 5'-AAA ATG GAA GCA TTT GGT AAT CA-3' (forward)
  5'-AGT GAA CTA AAA TTC TTA GAG GG-3' (reverse)
Exon 4: 5'-AAG CAA TGA TGA CAA AGT GCT AAC-3' (forward)
  5'-TGG TCC ACA ATG TCA CAT TTC GG-3' (reverse)
Exon 5a: 5'-CTG AAG GGC TGT GCT GTG GA-3' (forward)
  5'-TGT CCT TAC AAA CGG GGC T-3' (reverse).

### Multiplex Ligation Polymerase Assay (MLPA)

MLPA was used to detect large deletions in the *FXN* gene. The General Protocol issued by the manufacturer was followed (MRC-Holland, Amsterdam, Netherlands; version MDP-v002, 23.1.2012). The commercially available SALSA MLPA P136-B2 Recessive Ataxias Probemix was used (MRC-Holland, Amsterdam, Netherlands; lot 0511), which contains probes against the five coding exons in the *FXN* gene. DNA was first denatured, then hybridised to specific probes, which were then ligated. Fragment length analysis was undertaken on an ABI (Carlsbad, CA) 3730xl automated sequencer. The results were analysed on Genemarker, where the relative sizes of the fluorescent peaks within each sample were compared to reference samples.

## Results

### Genetic Analysis

**Figure 2:**
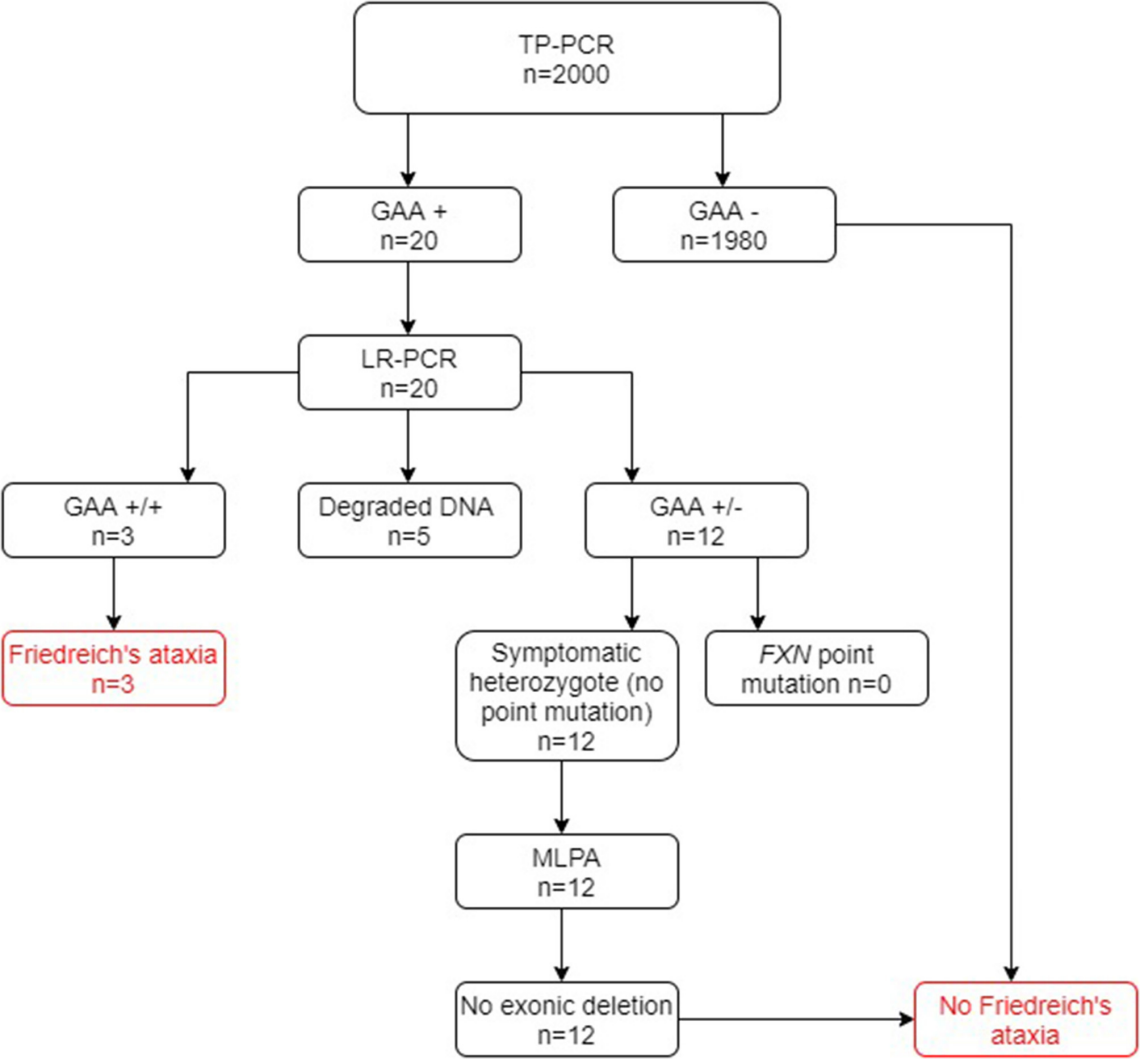
Flowchart summary of TP-PCR and Long PCR results in group A. Out of 2,000 patients, 20 patients tested positive for presence of GAA repeats by the TP-PCR method. Out of these 20 patients, only 3 tested FRDA-positive at the Long PCR stage for GAA expansions, with 12 being confirmed as carriers and the remaining 5 giving no result. Of the 12 FRDA carriers, none tested positive for *FXN* point mutations or exonic deletions. GAA+/+ two GAA expansions; GAA+/− one GAA expansion; GAA−/− no GAA expansions. MLPA, multiplex ligand-dependent probe amplification.

Of the 2000 cases in group A screened for FRDA, 20 cases tested positive at the TP-PCR stage, which confirmed the presence of a *FXN* GAA expansion on at least one allele. Of these cases, three were shown to have triplet repeat expansions on both alleles after LR-PCR and were thus newly diagnosed as being FRDA-positive (Figure 2). A further 12 cases were shown to have a *FXN* GAA expansion on only one allele and were therefore referred for testing for *FXN* point mutations and exonic deletions. Sequencing for point mutations was negative, and the MLPA assay indicated absence of exonic deletions in the *FXN* gene in each case. They were therefore confirmed as heterozygous FRDA carriers. Thus, 0.15% were newly diagnosed as FRDA-positive, and 0.6% were heterozygous carriers. Finally, five cases showed no result after gel or capillary electrophoresis, due very probably to DNA degradation.

**Figure 3:**
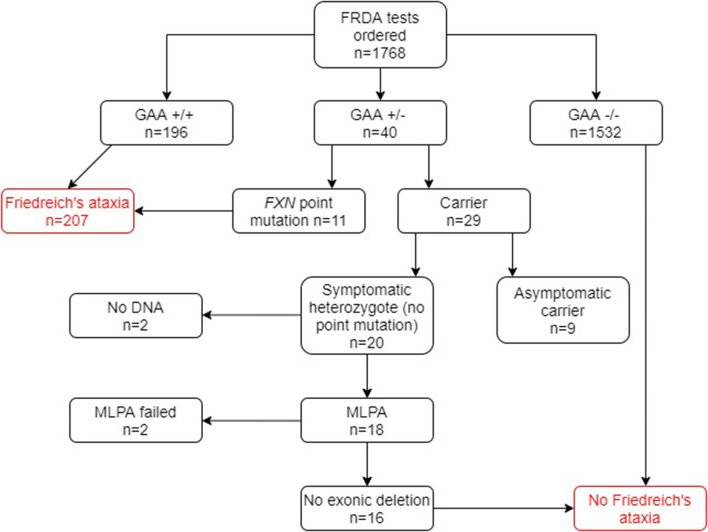
Flowchart summary of genetic results in group B patient cohort. Out of 1,768 patients, 207 patients tested positive for FRDA. GAA+/+ two GAA expansions; GAA+/− one GAA expansion; GAA−/− no GAA expansions. MLPA, multiplex ligand-dependent probe amplification.

**Figure 4:**
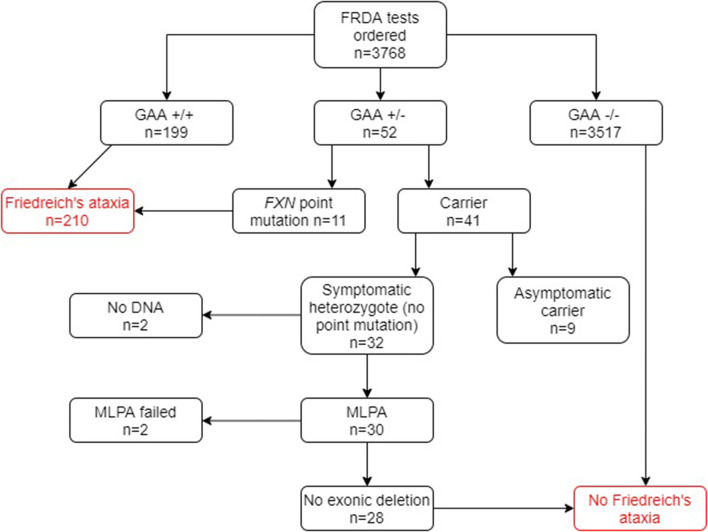
Flowchart summary of genetic results in both groups A and B combined. GAA+/+ two GAA expansions; GAA+/− one GAA expansion; GAA−/− no GAA expansions. MLPA, multiplex ligand-dependent probe amplification.

Of the 1,768 genetically undiagnosed ataxia patients who had been referred for a FRDA test (group B), 207 (11.7%) tested positive for FRDA (Figure 3). Eleven FRDA patients tested positive for compound heterozygosity with point mutations. The percentage of these among the FRDA-positive cases was 5.3%, which is greater than previously reported (4). A further 29 cases had one *FXN* expansion but no point mutation. Further study revealed that nine of these were asymptomatic and had been referred to the laboratory usually for predictive testing of unaffected relatives. Twenty affected cases with heterozygous *FXN* expansions therefore remained for further analysis by MLPA. No DNA was available for two cases and the MLPA failed in a further two cases. Of the remaining 16 cases, none had a large exonic deletion on MLPA. The heterozygous carrier rate was 1.6%. The results of the two groups combined are given in Figure 4.

### Clinical Features of FRDA Patients in Group A

The available clinical notes and medical letters of the three FRDA-positive cases discovered in group A were assessed and the findings are summarised in Tables 1, 2. All three had late onset with atypical clinical features. The mean±SD age at onset was 43.3 ± 16.1, with a range of 25–55. Patients 1 and 2, both male, had VLOFA, with ages at onset of 55 and 50 respectively. Neither patient had any family history of ataxia. The GAA expansion sizes for each patient in both alleles were very near to the minimum diagnostic threshold for FRDA (Patient 1, 105 GAA repeats; Patient 2, 120 GAA repeats). The first clinical symptom in patients 1 and 2 was gait ataxia.

**Table 1:** Summary of the age at onset, GAA triplet repeat expansion sizes and clinical features for all FRDA positive samples in group A.

**Patient no**.	**Age at onset**	**Expansion size, allele 1**	**Expansion size, allele 2**	**Summary of symptoms**
1	55	105	105	Progressive gait ataxia, brisk lower limb reflexes, extensor plantars, dysarthria, reduced vibration sense
2	50	120	120	Cerebellar ataxia, very slowly progressive gait and truncal ataxia, mild dysarthria, impaired balance
3	25	170	420	Progressive gait ataxia, impaired balance, paresthesias in hands and feet, areflexia, reduced vibration and joint position sense, mild dysarthria, blackouts

**Table 2:** Clinical features of FRDA-positive cases from group A.

**Patient no**.	**Ataxia**	**Tone**	**Pyramidal weakness**	**UL weakness**	**LL weakness**	**Areflexia**	**Extensor plantar response**	**Touch sensation loss**	**Vibration/positional loss**	**Dysarthria**	**Polyneuro-pathy**	**Nystagmus**	**SWJ**	**Optic atrophy**	**Diabetes**
1	Y	Normal	Y	N	Y	N	Y	N	Y	Y	N/R	Y	N	N	N
2	Y	Normal	N	N	N	N	N/R	N	N	Y	N	N	N	N	N
3	Y	N/R	N/R	N	N	Y	N/R	Y	Y	Y	Y	N	N	N	N

*Y, Yes; N, No; N/R, No Result; UL, Upper Limb; LL, Lower Limb; SWJ, Square-wave jerks*.

Patient 1 had progressive balance problems, incoordination, dysarthria and dysphagia. The original diagnosis was that of alcohol-induced ataxia due to his regular consumption of up to 100 units of alcohol a week for 30 years of his life.

Patient 2 had mild dysarthria and retained reflexes with a relatively pure cerebellar ataxia rather than the mixed cerebellar and sensory ataxia more typical of FRDA. Overall, patient 1 showed the more severe phenotype of the two patients, with pyramidal signs, reduced vibration sense in the legs, upgoing plantar reflexes and nystagmus, which were all absent in patient 2. There was no sign in either patient of non-neurological FRDA symptoms, such as pes cavus and cardiomyopathy.

Patient 3, a female patient, had LOFA, and was referred at age 27 for a progressive sensory neuropathy, and had also suffered from blackouts since age 17. At presentation, she had signs of mixed sensory and cerebellar ataxia, with wide-based ataxic gait, impairment of balance, mild dysarthria and gradually progressive numbness and paraesthesiae in her hands and feet. Unlike patients 1 and 2, patient 3 had areflexia and reduced joint position sense in the knees and elbows, and became wheelchair-bound in her early 30s. Her GAA expansion sizes were 170 and 420 which may account for her more severe phenotype and earlier age at onset. There was no family history of ataxia but one maternal uncle was diagnosed with multiple sclerosis in his 20s.

### Clinical Features of FRDA Patients in Group B

The clinical features of the FRDA patients in group B were assessed either from the medical notes or from their participation in a natural history study, the European Friedreich's Ataxia Consortium for Translational Studies (EFACTS). Eighty of the patients were in this study for whom systematic clinical data were recorded. Clinical data were also available for 26 other patients not in EFACTS. The clinical features of these 106 patients are summarised in Table 3. The mean ± SD age at onset was 14.3 ± 10.5 (range 1 to 51). The great majority of the patients had vibrational sensory loss (99.0%), areflexia (96.2%) and dysarthria (92.4%), with a slightly lower percentage having weakness (88.7%), square wave jerks (81.6%), extensor plantar reflexes (73.8%) and dysphagia (72.5%). Thus, the clinical features were more typical of classical FRDA compared to the three LOFA/VLOFA positive cases from group A.

**Table 3:** Clinical Features in FRDA Patients, group B.

**Feature**	**No**.	**%**	***n***
Paresis	94	88.7	106
Spasticity	54	55.1	98
Areflexia	100	96.2	104
Extensor plantars	76	73.8	103
Vibrational sensory loss	98	99	99
Square wave jerks	84	81.6	103
Nystagmus	59	57.3	103
Diabetes	10	9.5	105
Cardiomyopathy	59	56.7	104
Dysarthria	97	92.4	105
Dysphagia	74	72.5	102
Wheelchair-bound	72	67.9	106

## Discussion

The classical definition of the FRDA phenotype has gradually become more flexible due to multiple cases of atypical FRDA in the literature, including cases of late or very late onset (11, 12). Establishing a genetic diagnosis is essential for patient management, enabling counselling of patients and their relatives, and providing invaluable prognostic information. We present data on FRDA screening of 2000 SCA-negative ataxia patients who had not been previously screened for FRDA (group A). This is the largest screening study of its kind ever performed. We present clinical data from the three FRDA-positive patients. We also compared the results from this cohort with those from another cohort of patients who were referred for FRDA by their clinicians, in which many more FRDA cases and more typical presentations of the disease were expected to be found (group B).

In group B, 207 out of 1,768 (11.7%) tested FRDA-positive, with a heterozygous carrier rate of 1 in 63 (1.6%). Among group A however, only 3 out of 2,000 (0.15%) tested FRDA-positive. In combination with the cohort of ataxia patients who had not been referred, this gave a FRDA positive rate of 5.6% (210/3768) and a heterozygous carrier rate of 1.1%, well within the expected UK average (41/3768; Figure 4). As might be expected, patients who had been referred for a FRDA test had on average a more classical FRDA phenotype than the 3 FRDA patients in the other cohort, who had an atypical phenotype (Tables 1, 2). Mean age at onset in the FRDA-positive patients in group B was of 14.3 ± 10.5 (range 1 to 51), typical of classical FRDA in the majority of patients. This was significantly younger than the mean ± SD age at onset for the 3 FRDA-positive patients in group A of 43.3 ± 16.1 (range 25 to 55), indicating LOFA/VLOFA. Early symptoms in the cohort with classical FRDA included limb and truncal ataxia, in association with lower limb absent tendon reflexes. This was invariably followed by dysarthria, loss of vibration and joint position sense. There was also lower limb pyramidal tract dysfunction at some point over the next five years. Non-neurological symptoms such as cardiomyopathy and scoliosis were usually present: others, such as pes cavus, optic atrophy and nystagmus, were infrequent, which conformed with previous population studies (26). No point mutations or exonic deletions were detected in any of the 12 samples that displayed only one expanded GAA allele. Clinical information for the three FRDA-positive cases revealed an atypical phenotype in all three cases, and highly delayed-onset in two.

This study confirms that a diagnosis of FRDA is extremely rare in individuals with late onset and atypical features which would not usually prompt referral for FRDA testing. Such cases, however, do exist and have been previously reported, both before and after the discovery of the genetic basis for FRDA (26, 29, 30). The implications of this finding in terms of defining a genetic testing strategy may however depend on the healthcare system and access to whole genome sequencing (WGS). In healthcare systems in which WGS is readily available, a reasonable approach is to refer the patient for WGS after the common SCA expansion mutations have been excluded for patients without classical FRDA. WGS is able to detect triplet repeat expansions with increasing confidence (34) which may therefore still permit the identification of the very few patients with atypical FRDA. From an economic point of view, testing of FRDA in the 99.8% of patients who turn out to be negative is a greater cost burden than the 0.2% of patients in whom two *FXN* expansions may be detected by WGS. Alternatively direct referral for WGS with no prior expansion screening may also be considered, which is likely to be the approach taken in the future for genetic diagnostic testing. In countries or healthcare settings with limited or no access to WGS, a different approach which includes screening for *FXN* expansions in all patients may be necessary in order to ensure patients receive the most comprehensive screening possible within the confines of the setting.

The three cases of FRDA identified from group A may provide some information about when FRDA testing is indicated in atypical cases, despite some limitations to the study such as the unavailability of MRI scans of participants. None of the three cases came from specialist centres for ataxia, but rather from general neurologists. In patient 1, there was a prominent history of excess alcohol consumption amounting to some 100 units per week for 30 years which undoubtedly will have contributed to the development of ataxia. However, the patient's cerebellar ataxia was progressive and did not remit upon cessation of alcohol consumption. Although alcoholic ataxia can progress after cessation of alcohol consumption (35), we recommend that patients with alcohol-related ataxia whose disease continues to progress despite abstinence should be tested for potential underlying genetic syndromes such as FRDA.

Patient 2 displayed a pure cerebellar ataxia, which is uncharacteristic of FRDA. He also had very late onset of disease. His brother, who was tentatively diagnosed with multiple sclerosis in his mid-20s due to progressive deterioration of motor function and movement leading to wheelchair use in his 30s, may well also have had FRDA. If so, the 25-year gap in age at onset between the brothers is particularly striking. GAA expansion instability is known to occur in transmission from parent to child (36). This case may highlight the potential scope of GAA expansion instability in an intrafamilial context and highlights the difficulty that can be involved in making clinical diagnoses. Consistent collection of family history and direct assessment of affected family members may help avoid missing atypical genetic cases.

The GAA expansion sizes for the first two patients on both alleles were very near the minimum diagnostic threshold for FRDA (Patient 1, 105 GAA repeats; Patient 2, 120 GAA repeats), which is also very likely to be linked to the very late onset, slow progression and atypical features in both patients.

Finally, patient 3 had only borderline late onset at age 25. Despite a progressive neuropathy and a wide-based ataxic gait, an early diagnosis of sensory axonal peripheral neuropathy was made and she was not tested for FRDA. A later nerve biopsy showed no evidence of inflammatory change in the sural nerve. Careful examination should look for the combination of cerebellar and neuropathic features as seen in FRDA. The patient also experienced blackouts from a standing position of ten minutes each from age 17. Syncope has been linked to cardiac arrhythmia, which often occurs in the late stages of FRDA (11). Cardiac investigations such as echocardiogram and prolonged ECG monitoring may also help inform the diagnosis of FRDA.

Among the clinical phenotypes of the heterozygous FRDA carriers available (9 out of 12), none showed specific symptoms that indicated underlying FRDA (data not shown). All patients are assumed to have unrelated ataxias, on which their single GAA triplet repeat expansion has no or minimal influence, although it is also possible that some as yet undetected genetic basis for FRDA is the cause of their ataxia. This is the largest data set for which an estimate of carrier frequency for FRDA has been attempted in the UK. In group A, we report 12 heterozygous FRDA carriers out of a total of 2,000 patients, giving a 1:165 FRDA carrier frequency in this ataxic population. This is considerably lower than the previously reported FRDA carrier frequency in western European populations of between 1:60 and 1:110. The FRDA carrier frequency in group B is instead consistent at 1:65 (25, 37). The carrier rate obtained when combining the two cohorts was of 41/3768 or around 1:90, and is consistent with previous reports. The negative results from the point mutation and exonic deletion testing of the 12 FRDA carriers were expected, given the rarity of these mutations (and extreme rarity in the case of exonic deletions) compared with the expansion in FRDA cases.

This study only evaluates those genetic changes which have robustly been shown to be associated with clinical cases of Friedreich's ataxia and are currently routinely used in clinical practise, namely GAA expansions, point mutations and large exonic deletions. This study did not evaluate non-coding variants (other than the intronic GAA expansion) including promoter and regulatory regions of the gene, epigenetic variants, frataxin expression levels or other mechanisms which may yet explain some of the remaining cases but which are beyond the scope of this study (19, 38, 39). It is expected that increased testing of patients by WGS will result in an increase in the number of patients in whom a clinical diagnosis can be confirmed as well as expanding the genotype-phenotype correlation for known genes. For this reason, we recommend referral of such cases to specialist genetics or ataxia centres where greater multidisciplinary clinical and genetic experience and more extensive genetic testing are available, depending on the resources of the local healthcare system.

## Conclusions

This is the largest screening study of patients referred for testing of genetic causes of ataxia, and assesses the frequency of *FXN* GAA expansions, point mutations and exonic deletions in patients referred to a tertiary hospital specialist neurogenetics laboratory (at the National Hospital for Neurology and Neurosurgery, London, UK). The study shows that in cases referred for testing for FRDA, the positive cases have a typical phenotype. Amongst the group in whom only SCAs rather than FRDA were suspected, atypical cases of FRDA can still occur, but the frequency is extremely low. In these cases, a referral to a specialist ataxia centre is warranted where more extensive testing can be performed and interpreted in a multidisciplinary setting to ensure appropriate patient management and treatment.

## Data Availability Statement

The raw data supporting the conclusions of this article will be made available by the authors, without undue reservation.

## Ethics Statement

The studies involving human participants were reviewed and approved by UCLH Joint Research Office, approved as a Service Evaluation. Written informed consent for participation was not required for this study in accordance with the national legislation and the institutional requirements.

## Author Contributions

AB and MP undertook all laboratory work and wrote the original manuscript. MP, HG-M, and PG assessed patients clinically and reviewed previous clinical notes. EM, RL, and MS identified patients and contributed to laboratory work. PG conceived and supervised the project. All authors have contributed to review the manuscript.

## Funding

PG was supported by the National Institute for Health Research University College London Hospitals Biomedical Research Centre UCLH and also from the North Thames CRN. PG, MP, and HG-M work at University College London Hospitals/University College London, which receives a proportion of funding from the Department of Health's National Institute for Health Research Biomedical Research Centre's funding scheme. Funded by UCL Grand Challenges Scheme, National Brain Appeal Small Acorns Fund, Ataxia UK, European Friedreich's Ataxia Consortium for Translational Studies (EFACTS), and UK National Institute of Health Research (NIHR).

## Conflict of Interest

The authors declare that the research was conducted in the absence of any commercial or financial relationships that could be construed as a potential conflict of interest.

## Publisher's Note

All claims expressed in this article are solely those of the authors and do not necessarily represent those of their affiliated organizations, or those of the publisher, the editors and the reviewers. Any product that may be evaluated in this article, or claim that may be made by its manufacturer, is not guaranteed or endorsed by the publisher.

## Abbreviations

FRDA: Friedreich's ataxia
ILOCA: Idiopathic late-onset cerebellar ataxia
LOFA: Late-onset Friedreich's ataxia
Fe-S clusters: Iron-sulphur clusters
PCR: Polymerase chain reaction
TP-PCR: Triplet-repeat primed PCR
SCA: Spinocerebellar ataxia
MLPA: Multiplex ligation-dependent probe amplification assay
WES: Whole Exome Sequencing
WGS: Whole Genome Sequencing.
